# Editorial: Neuroimmunology of the Inner Ear

**DOI:** 10.3389/fneur.2021.635359

**Published:** 2021-02-09

**Authors:** Paola Perin, Franca Marino, Isabel Varela-Nieto, Agnieszka J. Szczepek

**Affiliations:** ^1^Department of Brain and Behavioural Sciences, University of Pavia, Pavia, Italy; ^2^Center of Research in Medical Pharmacology University of Insubria, Varese, Italy; ^3^Institute for Biomedical Research “Alberto Sols” (IIBM), Spanish National Research Council-Autonomous University of Madrid (CSIC-UAM), Madrid, Spain; ^4^Rare Diseases Networking Biomedical Research Centre, Centro de Investigación Biomédica en Red, Carlos III Institute of Health, Madrid, Spain; ^5^La Paz Hospital Institute for Health Research (IdiPAZ), Madrid, Spain; ^6^Department of Otorhinolaryngology, Head and Neck Surgery, Charité-Universitätsmedizin Berlin, Corporate Member of Freie Universität Berlin, Humboldt-Universität zu Berlin, and Berlin Institute of Health, Berlin, Germany; ^7^Faculty of Medicine and Health Sciences, University of Zielona Gora, Zielona Gora, Poland

**Keywords:** inner ear, immunology, neuroimmunology, balance, hearing, disorders

Although the term was first officially used in 1982 (1), neuroimmunology is now a mature field that has gained immense traction in the past decade. Thanks to novel technological advances, the cellular and molecular mechanisms that mediate the crosstalk between the immune and nervous systems are increasingly appreciated in both physiological and pathological states (1).

Similar to the brain, the inner ear has long been considered an “isolated” system devoted to auditory and vestibular signal processing and protected by a blood-labyrinth barrier (BLB) (2–5), and the early neuroimmunology of the inner ear was mainly focused on autoimmunity (6, 7), and on the role of macrophages in cochlear damage (8, 9). In parallel to the brain, awareness about non-neural cells and molecules affecting inner ear functions has been steadily growing^1^, and neuro-immunological studies of the inner ear face multiple challenges, including an overwhelming number of cellular and molecular interactions, which will require a systems biology approach to grasp their full functionality. In addition, the inner ear poses unique difficulties due to its tight bone encasing and complex fluid regulation.

Like most organs, including the brain, the inner ear immune cells are dominated by several populations of macrophages [reviewed in (10, 11)], which largely contribute to both inflammatory/phagocytic and regenerative/protective responses. However, several questions are still open, such as:

- What are the signals exchanged between immune cells and inner ear cells in healthy and pathological settings?
- How much communication is there between the inner ear and surrounding tissues and fluids?
- What is the neuroimmune role of the endolymphatic sac?
- What are the roles, nature, and location of several immune cell populations and subpopulations, e.g., mast cells (12), lymphocytes (13), or other leukocytes (14)?
- How are local and systemic immune responses regulated—and especially dysregulated- in various kinds of damage (e.g., infection, noise trauma, and ototoxicity)?
- How do neuroimmune interactions translate in the modulation of inner ear functions?

The articles collected in this Research Topic reflect this increasingly diverse field in several main threads, broadly divided into immune cell characterization, responses to diseases or damage, biomarkers, and immune molecular targets (Figure 1). Both human and animal studies are included in this Special Topic. The system's complexity at both cellular and functional levels requires the integration of invasive approaches only possible in animals and testing of functions and markers that may be incompletely overlapping between humans and other animals. Table 1 summarizes the main points that were contributed by human and animal studies within this Research Topic.

**Figure 1:**
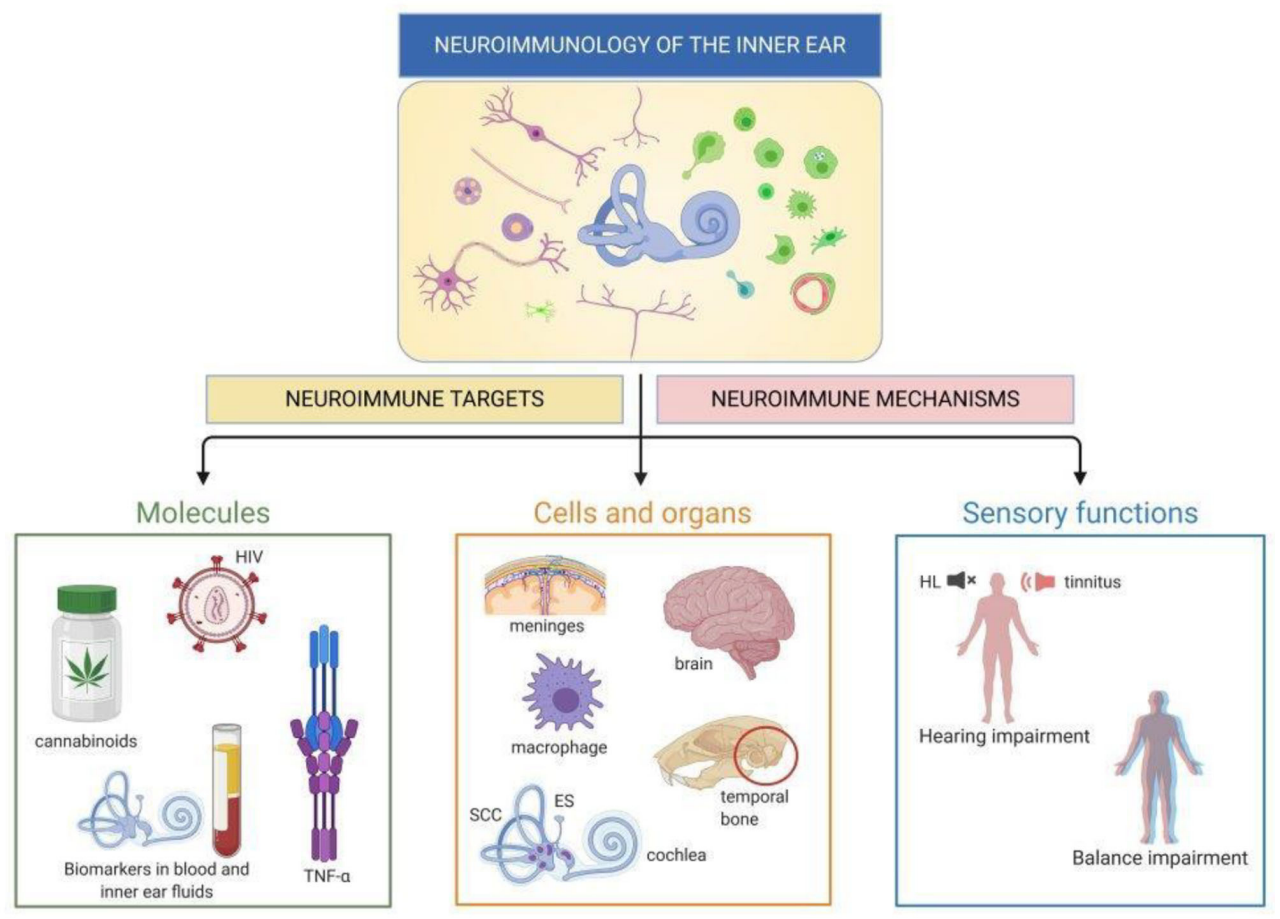
Neuroimmunology of the inner ear. Visual abstract of this Research Topic: each paper within this Topic explores molecules, cells and/or functions related to inner ear neuroimmune interactions. HL, hearing loss; ES, endolymphatic sac; SCC, semicircular canals. Created with Biorender.com.

**Table 1:** A summary of data published in the Research Topic “Neuroimmunology of the Inner Ear” based on the type of manuscript and the research model used.

**References**	**Type of manuscript**	**Discovery**	**Model**
**ANIMAL STUDIES**			
“Early Development of Resident Macrophages in the Mouse Cochlea Depends on Yolk Sac Hematopoiesis” (Kishimoto et al.)	Original Research	Two resident macrophage populations derived from the yolk sac and fetal liver	Mouse
“Csf1 Signaling Regulates Maintenance of Resident Macrophages and Bone Formation in the Mouse Cochlea” (Okano and Kishimoto)	Original Research	The influence of Csf1op/op genotype (where no Csf1 is produced) on cochlear bone remodeling and cochlear macrophages, pointing to a possible connection between immune responses and bony capsule metabolism	Mouse
“Lack of Fractalkine Receptor on Macrophages Impairs Spontaneous Recovery of Ribbon Synapses After Moderate Noise Trauma in C57BL/6 Mice” (Kaur et al.)	Original Research	The negative impact of knocking-out the fractalkine receptor on ribbon synapse recovery after noise trauma - a protective role for auditory nerve-associated macrophages	Mouse
“Anti-inflammatory and Oto-Protective Effect of the Small Heat Shock Protein Alpha B-Crystallin (HspB5) in Experimental Pneumococcal Meningitis” (Erni et al.)	Original Research	Protective effect of alpha B-crystallin (HspB5) on the inner ear damage due to meningococcal infection	Rat
“Immune Response After Cochlear Implantation” (Simoni et al.)	Original Research	Dexamethasone-eluting electrodes reduce the inflammatory response induced by cochlear implantation	Guinea pig
“Cytokine Levels in Inner Ear Fluid of Young and Aged Mice as Molecular Biomarkers of Noise-Induced Hearing Loss” (Landegger et al.)	Original Research	Discovered reduced concentrations of IL-1β and TNF-α in the perilymph of 2-year-old mice compared to adolescent mice. Discovered that exposure to noise associates with increased levels of the chemokine (C-X-C motif) ligand 1, IL-6 and TNF-α in the perilymph of adolescent mice.	Mouse
“Intracochlear Perfusion of Tumor Necrosis Factor-Alpha Induces Sensorineural Hearing Loss and Synaptic Degeneration in Guinea Pigs” (Katsumi et al.)	Original Research	TNF-α perfusion rapidly induced synaptic loss and CAP reduction	Guinea pig
“Nfatc4 Deficiency Attenuates Ototoxicity by Suppressing Tnf-Mediated Hair Cell Apoptosis in the Mouse Cochlea” (Zhang et al.)	Original Research	Expression of Nfatc4 in the auditory hair cells associates with TNF-dependent apoptosis	Mouse
**CLINICAL STUDIES**			
“Vertigo and Severe Balance Instability as Symptoms of Lyme Disease—Literature Review and Case Report” (Jozefowicz-Korczynska et al.)	Case report and literature review	The effects of neuroborreliosis on the human vestibular system	Human
“Age-Related Changes in Immune Cells of the Human Cochlea” (Noble et al.)	Original Research	Macrophage populations associated with various parts of the human cochlea in respect of aging	Human (cochlea)
“Human Inner Ear Immune Activity: A Super-Resolution Immunohistochemistry Study” (Liu et al.)	Original Research	Macrophages and T lymphocytes in the inner ear	Human (cochlea and endolymphatic sac)
“Early Diagnosis of Hearing Loss in Patients Under Methadone Maintenance Treatment” (Bayat et al.)	Original Research	Mechanisms of ototoxicity secondary to methadone treatment	Human
“Subcellular Abnormalities of Vestibular Nerve Morphology in Patients with Intractable Menière's Disease” (Wang et al.)	Original Research	Presence of structures associated to neurodegeneration in the vestibular nerve of Meniere‘s disease patients	Human
“Differential Proinflammatory Signature in Vestibular Migraine and Meniere Disease” (Flook et al.)	Original Research	Cytokine expression pattern from peripheral blood mononuclear cells discriminating between healthy controls, vestibular migraine patients, and two subgroups of Meniere‘s disease	Human
“Defining the Inflammatory Microenvironment in the Human Cochlea by Liquid Biopsy and Perilymph Analysis” (Warnecke et al.)	Original Research	Analysis of human perilymph obtained during cochlear implantation demonstrated feasibility of method and identified potential biomarkers differentiating between the patients with residual hearing and complete deafness	Human
“Correlations Between Vestibular Function and Imaging of the Semicircular Canals in DFNA9 Patients” (Ihtijarevic et al.)	Original Research	Demonstrated and association between CT and MRI abnormalities in DFNA9 patients carrying the P51S mutation in the gene encoding cochlin	Human
**REVIEWS (CLINICAL STUDIES)**			
“Biomarkers in Vestibular Schwannoma–Associated Hearing Loss” (Lassaletta et al.)	Mini Review	Synthesis of knowledge about biomarkers characterizing sporadic vestibular schwannoma and VS associated with neurofibromatosis type 2	Human
“Main Aspects of Peripheral and Central Hearing System Involvement in Unexplained HIV-Related Hearing Complaints” (de Jong et al.).	Review	auditory consequences of HIV viral infection	Human
**REVIEWS (CLINICAL AND ANIMAL STUDIES)**			
“Genetic Hearing Loss Associated With Autoinflammation” (Nakanishi et al.)	Review	Review of evidence associating the NLRP3 inflammasome with deafness	Human, mouse
“Cannabinoids, inner ear, hearing and tinnitus: a neuroimmunological perspective” (Perin et al.).	Review	Review of effects of cannabinoid on the brain-related and ear-related immune system with emphasis on tinnitus and hearing loss	Human, mouse, rat

Five papers published in this Research Topic focus on the description of the immune cells of the inner ear, with a predominance of studies regarding cochlear macrophages, consistent with their role as leading players both by number and function, as reviewed in (10, 11).

The paper “Early Development of Resident Macrophages in the Mouse Cochlea Depends on Yolk Sac Hematopoiesis” describes the presence of two resident macrophage populations in the mouse cochlea, derived respectively from the yolk sac and fetal liver (Kishimoto et al.). These populations are different in their Csf1-dependence and final cochlear localization. The origin of macrophages may be reflected in their various shapes in the adult cochlea, as seen in human samples in the following two papers. The first, “Age-Related Changes in Immune Cells of the Human Cochlea,” describes macrophage populations associated with various parts of the human cochlea and the aging-dependent changes occurring in these cells (Noble et al.). Similarly, the paper “Human Inner Ear Immune Activity: A Super-Resolution Immunohistochemistry Study,” describes immune cells (mainly macrophages and to a lesser extent T lymphocytes) associated with the human cochlea and endolymphatic sac (Liu et al.). Both papers observed an association between cell shapes and their localization in the inner ear. Finally, two groups studied macrophage-related gene knock-out effects on the inner ear function and anatomy using the mouse model. “Csf1 Signaling Regulates Maintenance of Resident Macrophages and Bone Formation in the Mouse Cochlea” underlines the impact of the Csf1op/op genotype (where no Csf1 is produced) on cochlear bone remodeling and cochlear macrophages, therefore pointing to a possible connection between immune responses and bony capsule metabolism (Okano and Kishimoto). On the other hand, the paper “Lack of Fractalkine Receptor on Macrophages Impairs Spontaneous Recovery of Ribbon Synapses After Moderate Noise Trauma in C57BL/6 Mice” demonstrates the negative impact of knocking-out the fractalkine receptor on ribbon synapse recovery after noise trauma, suggesting a protective role for auditory nerve-associated macrophages (Kaur et al.).

Eight other papers in this Research Topic studied immune responses in the inner ear in a pathological context, three of which (two in humans and one in a rat model) focus on infectious diseases. Passive barriers strongly protect the inner ear, but pathogens may enter it through its connections with CSF and middle ear, plus vascular and neural routes additionally available for viruses (15). Moreover, even systemic responses to pathogens may affect the ear indirectly, due to immune crossreactivity (as suggested after viral or fungal infection, reviewed in (16) or BLB impairment opening the way to ototoxic factors into the inner ear (17–19).

The review “Main Aspects of Peripheral and Central Hearing System Involvement in Unexplained HIV-Related Hearing Complaints” presents an interesting study on the auditory consequences of HIV viral infection (which show similarities to age-induced hearing impairment), an aspect not well-understood and of enormous importance (de Jong et al.). The paper “Vertigo and Severe Balance Instability as Symptoms of Lyme Disease—Literature Review and Case Report” describes the effects of neuroborreliosis, which can target the 8th nerve, on the human vestibular system from a clinical point of view (Jozefowicz-Korczynska et al.). The work described in “Anti-inflammatory and Oto-Protective Effect of the Small Heat Shock Protein Alpha B-Crystallin (HspB5) in Experimental Pneumococcal Meningitis” demonstrates in a rat model that inner ear damage due to meningococcal infection goes together with an increase of proinflammatory cytokines in CSF, and rise in the numbers of cochlear neutrophils and macrophages (Erni et al.). The cytokine (but not the cellular) response could be reduced by intracisternal injection with the small heat shock protein alpha B-crystallin (HspB5), which also ameliorated hearing loss.

Besides infections, the inner ear can be affected by exposure to stress factors (including noise, drugs, and surgery), aging, genetic defects, or pathologies of unclear etiology, such as Menière's disease (MD). Many of these settings are accompanied by inflammation—a classical response to damage that is beneficial *per se* but may become detrimental if dysregulated, further damaging the inner ear [reviewed in (20, 21)]. Moreover, inflammation related to invasive inner ear intervention, such as cochlear implantation (CI), may lead to fibrosis and bone neoformation (22, 23), which degrade residual hearing. An effective otoprotective strategy in CI is the use of dexamethasone-eluting electrodes (24–26). However, understanding the otoprotective mechanisms of dexamethasone in CI is difficult since steroids can block all inflammatory response phases (20). Interestingly, CI influences the composition of macrophages subsets in animals (27, 28) and humans (29, 30). In the animal model, where the entire inflammatory process can be followed, macrophages have been found in the inflammatory, cytotoxic phenotype (27), and in the reparative phenotype, which is associated to matrix deposition and remodeling, and therefore to fibrosis (28). Moreover, a protective macrophage subpopulation has been observed in rodents' spiral ganglion (31) and suggested to exist in humans (29). The foreign-body responses may also induce the macrophages to form giant multinucleated cells with osteoblast-like properties (32). Finally, CI may also recruit B and T lymphocytes (33). The manuscript “Immune Response After Cochlear Implantation” shows that dexamethasone-eluting electrodes reduce both the cellular and cytokine signature of acute inflammatory response and fibrosis associated with implantation in a guinea pig, a well-established animal model for CI (Simoni et al.).

The paper “Genetic Hearing Loss Associated With Autoinflammation” describes deafness correlated with mutations affecting the NLRP3 inflammasome and other genes that influence macrophages (Nakanishi et al.). Inflammation of strial blood vessels was suggested, among other possible causes, in ototoxicity secondary to methadone treatment in humans in the paper “Early Diagnosis of Hearing Loss in Patients Under Methadone Maintenance Treatment” by Bayat et al..

Finally, two papers focus on the immune-inflammatory response in Menière's disease. As of today, MD diagnosis and monitoring are based on clinical symptoms, and no selective biomarker is available (34, 35). Endolymphatic hydrops has been long associated (although not exclusively) with MD, and endolymph-producing and reabsorbing structures in the ear are being targeted in treatment options [reviewed in (34, 35)]. Also, recent studies show a breakdown of the human utricular BLB in MD due to increased vesicular transport in endothelial cells and pericytes (36). Therefore, perilymph production appears to be affected, as well. In fact, a diagnostic tool based on gadolinium-enhanced MRI is being considered for MD (37, 38).

MD etiology is still debated and includes a combination of genetic, immune, inflammatory, environmental, hemodynamic, and hormonal factors (39). Immune-inflammatory responses seem however central, given that several genes linked to MD belong to immune or inflammation pathways (40), and that a significant percentage of patients with MD is also affected by other autoimmune diseases (41), or displays enhanced and anomalous inflammatory responses (42). Moreover, the endolymphatic sac, which is involved in endolymph reabsorption and displays morphological changes in MD (43), is the primary immune structure of the inner ear (44), and inner ear vasculature permeability is strongly affected by inflammation (45). Finally, MD immune-inflammatory model explains the effectiveness of steroid treatment (46). However, as for other immune diseases, treatment of subjects non-responsive to steroids requires personalized approaches that depend on the particular pathway being deranged (40).

The report “Subcellular Abnormalities of Vestibular Nerve Morphology in Patients with Intractable Menière's Disease” found the presence of structures associated to neurodegeneration (corpora amylacea, lipofuscin, microglia) in the vestibular nerve of MD patients (Wang et al.). On the other hand, the paper “Differential Proinflammatory Signature in Vestibular Migraine and Meniere Disease” describes a cytokine subset whose expression level in peripheral blood mononuclear cells can discriminate between healthy controls, vestibular migraine patients, and two subgroups of MD patients, thus yielding the basis for a blood test helping MD diagnosis (Flook et al.).

Two papers focused on a hot spot in auditory research: biomarkers for various types of inner ear diseases. “Cytokine Levels in Inner Ear Fluid of Young and Aged Mice as Molecular Biomarkers of Noise-Induced Hearing Loss” approaches the connection between noise-induced and age-related hearing loss using the mouse model (Landegger et al.). The second paper, “Biomarkers in Vestibular Schwannoma–Associated Hearing Loss,” reviews the proteins and genes that could potentially be included in the clinical evaluation panel of vestibular schwannoma (Lassaletta et al.).

The paper “Defining the Inflammatory Microenvironment in the Human Cochlea by Liquid Biopsy and Perilymph Analysis” studied human perilymph from patients assigned for CI, observing the expression of a panel of immune-related and growth factor-related proteins (Warnecke et al.). Finally, in the paper “Correlations Between Vestibular Function and Imaging of the Semicircular Canals in DFNA9 Patients,” MRI was used to visualize abnormalities of the semicircular canals that were attributed to cochlin deposits and fibrosis in the light of functional deficiencies (Ihtijarevic et al.).

Regarding molecular targets for neuroimmune signaling in the ear, most papers in this Research Topic confirmed an essential role for TNF signaling [see discussion in (47)]. Two papers focused on that issue. “Intracochlear Perfusion of Tumor Necrosis Factor-Alpha Induces Sensorineural Hearing Loss and Synaptic Degeneration in Guinea Pigs” found that TNF-α perfusion rapidly induced synaptic loss and CAP reduction, which resembled primary cochlear neuropathy (Katsumi et al.), whereas “Nfatc4 Deficiency Attenuates Ototoxicity by Suppressing Tnf-Mediated Hair Cell Apoptosis in the Mouse Cochlea” describes the expression of Nfatc4 in the auditory hair cells being linked to TNF-dependent apoptosis (Zhang et al.).

The last paper, “Cannabinoids, inner ear, hearing and tinnitus: a neuroimmunological perspective” reviews cannabinoid effects on the immune system and their possible roles in tinnitus and hearing loss, for which only neuronal effects have been considered so far (Perin et al.).

By exploring several consolidated or novel mechanisms and effects of neuroimmune interactions in the inner ear, this Research Topic yields a broad perspective on possible innovative therapeutic and diagnostic approaches to audiovestibular diseases and contributes to increasing visibility of this fascinating subject. We hope that in the next decade, the current research will elucidate biochemical pathways connecting immune responses in sensory circuits to functional changes at the cellular and systems levels. Further exploration of the inner ear's neuro-immuno-sensory axis might impact future therapy and monitoring of some otological diseases.

## Author Contributions

PP and AS drafted the manuscript. PP drew the figure. PP, FM, IV-N, and AS revised and approved the final version of this manuscript. All authors contributed to the article and approved the submitted version.

## Conflict of Interest

The authors declare that the research was conducted in the absence of any commercial or financial relationships that could be construed as a potential conflict of interest.

## Footnotes

^1^PUBMED search with the query “(inner ear OR cochlea OR vestibular) AND (neuroimmune OR neuroimmunology)” only retrieves 14 results, but this reflects the still sparse use of “neuroimmune”, since the related queries “(inner ear OR cochlea OR vestibular) AND (immune OR macrophage OR cytokine)” and “(inner ear OR cochlea OR vestibular) AND (inflammatory OR inflammation)” retrieve 1,734 and 2,043 results, respectively.
